# Cost-effectiveness analysis of dapagliflozin for the treatment of type 2 diabetes mellitus in Spain: results of the DECLARE-TIMI 58 study

**DOI:** 10.1186/s12913-022-07567-5

**Published:** 2022-02-17

**Authors:** Carlos Escobar, Cristóbal Morales, Margarita Capel, Susana Simón, Ferran Pérez-Alcántara, Elisenda Pomares

**Affiliations:** 1 Cardiology Department, Hospital La Paz, Madrid, Spain; 2 Endocrinology Department, Hospital Vithas Sevilla, Sevilla, Spain; 3 HEOR & Market Access, AstraZeneca, Madrid, Spain; 4 Oblikue Consulting S.L., Barcelona, Spain

**Keywords:** Cost-effectiveness analysis, Dapagliflozin, DECLARE, Type 2 diabetes mellitus, Spain

## Abstract

**Background:**

The objective of this study was to carry out a cost-effectiveness analysis of dapagliflozin, as an add-on therapy to standard of care (SoC), for the treatment of type 2 diabetes mellitus (T2DM) in Spain, based on the results of the DECLARE-TIMI 58 trial.

**Methods:**

A discrete event simulation model (Cardiff T2DM) based on the data observed in the DECLARE-TIMI 58 trial was adapted to the Spanish setting to estimate the costs and health outcomes of treatment with dapagliflozin in patients with T2DM who had or were at risk of atherosclerotic cardiovascular disease. Macrovascular events (hospitalization for heart failure, myocardial infarction, stroke, and unstable angina), end-stage renal disease and cardiovascular and non-cardiovascular mortality were modeled according to the survival equations of the DECLARE-TIMI 58 study. Microvascular events (blindness and ulcers) were estimated based on the risk equations of the UK Prospective Diabetes Study. The analysis was conducted from the Spanish National Health System perspective and the time horizon was 30 years. The results were evaluated in terms of cost per quality-adjusted life year (QALY) gained. Only direct health costs were included, and a 3% discount rate was applied to costs and health outcomes. Univariate and probabilistic sensitivity analyses (PSA) were made to assess the robustness of the results.

**Results:**

In the main analysis, dapagliflozin was a dominant therapeutic option compared with placebo, with greater effectiveness (0.08 QALYs) and lower associated total costs per patient (€ -2,921). The univariate sensitivity analysis and the PSA confirmed the robustness of the results. The PSA showed the probability that dapagliflozin was a dominant alternative compared with placebo was 84.2% and that it was cost-effective of 92.1%, under a willingness-to-pay of € 20,000/QALY gained.

**Conclusions:**

The analysis of data from the DECLARE-TIMI 58 trial shows that dapagliflozin would be a cost-effective option in Spain for the treatment of adult patients with T2DM, as an add-on therapy to SoC, compared with placebo.

**Supplementary Information:**

The online version contains supplementary material available at 10.1186/s12913-022-07567-5.

## Background

Diabetes mellitus (DM) is a chronic disease with a high socioeconomic impact due to its associated morbidity and mortality. Patients with DM have two to three times increased risk of cardiovascular morbidity than the general population [1]. In addition, the direct costs of DM account for between 8% and 13% of total health expenditure by the Spanish National Health System (NHS) and were estimated at € 5,809 million in 2012 [2, 3]. The main conditioning factors are the costs of hospitalization and pharmacological treatment, which account for more than 70% of direct costs (41% and 32%, respectively) [3]. Diabetes complications increase the pharmacological and disease management cost, as well as the risk of hospitalization, and involve significant productivity loss [3].

Type 2 DM (T2DM) accounts for around 90% of all cases of diabetes [4]. In Spain, the prevalence of T2DM is estimated at 13.8%, of which approximately 6% are undiagnosed [5], and the incidence rate is 11.6 cases per 1,000 person-years [6]. T2DM is characterized by hyperglycemia and caused by insufficient secretion of insulin from pancreatic beta cells and insulin resistance [7]. T2DM is often associated with obesity and other modifiable risk factors (sedentary lifestyle, smoking, diet, high blood pressure, dyslipidemia, etc.) that increase the cardiovascular risk and reduce the quality of life [1, 7].

Currently, the management of T2DM requires a multifactorial and individualized approach to control blood glucose and other risk factors [7]. The main recommendation to achieve glycemic control is lifestyle modification (physical activity and diet). However, when it is not sufficient, metformin remains the first choice of treatment for most patients [7].

In uncontrolled patients with T2DM, sodium-glucose cotransporter-2 inhibitors have proven efficacy in achieving sustained glycemic control, providing cardiovascular benefits, and reducing body weight and blood pressure, without increasing the hypoglycemic risk [8]. The DECLARE-TIMI 58 trial evaluated the effects of dapagliflozin on cardiovascular and renal outcomes in 17,160 T2DM patients who had or were at risk for atherosclerotic cardiovascular disease [9]. Participants were randomly assigned to dapagliflozin 10 mg/day or placebo, as an add-on therapy to standard of care (SoC). Treatment with dapagliflozin result in a lower rate of hospitalization for heart failure (HF) and cardiovascular death, and a reduction in the progression of kidney disease, compared with placebo [9].

In Spain, dapagliflozin is reimbursed in adults for the treatment of insufficiently controlled T2DM as an adjunct to diet and exercise as monotherapy when metformin is considered inappropriate due to intolerance, and in addition to other medicinal products for the treatment of T2DM. Dapagliflozin is prescribed by endocrinologists in the hospital setting.

 The objective of this study was to evaluate cost-effectiveness of dapagliflozin, as an add-on therapy to SoC, for the treatment of uncontrolled patients with T2DM in Spain, according to the results of the DECLARE-TIMI 58 trial.

## Methods

### Model structure and type of analysis

A cost-effectiveness analysis was used to evaluate the use of dapagliflozin, as an add-on therapy to SoC, compared with placebo for the treatment of T2DM from the NHS perspective. This analysis was performed by adapting the Cardiff T2DM Model, a Monte Carlo simulation model with individual fixed time increments that was developed using equations from the United Kingdom Prospective Diabetes Study [10–12], and was previously validated to simulate the disease progression of patients with T2DM [13–16]. This model has recently been updated to include the survival curves observed in the DECLARE-TIMI 58 trial [9, 17] and a module to track the progression of the estimated glomerular filtration rate (eGFR) through stages 2-5 of chronic kidney disease (CKD). Model predictions over a time horizon of 4.2 years were validated to results from the DECLARE-TIMI 58 trial (see Additional file 1). To further validate the model, the structure, main assumptions and inputs were validated with clinical experts to ensure it simulated clinical practice in Spain.

The model simulated the natural history of T2DM in a cohort of 1,000 patients, considering usual Spanish clinical practice, treatment effectiveness and direct healthcare costs. The costs associated with drug acquisition, treatment discontinuation, T2DM-related micro- and macrovascular complications, adverse events (AEs) and severe hypoglycemic events, and the management of CKD were included. Macrovascular events (hospitalization for HF, myocardial infarction, stroke, unstable angina), all-cause mortality and end-stage renal disease (ESRD) were predicted by specific survival curves of each event, which were fitted to Kaplan-Meier data collected over the DECLARE-TIMI 58 trial and extrapolated from 4-year survival curves over the time horizon. Spanish life tables were applied if the age- and gender-specific probability of mortality in the general population exceeded the predicted probability from the survival curves. Microvascular events (blindness and ulcers) were estimated based on UKPDS 82 study risk equations [11].

The simulations were performed individually for each patient in 6-month cycles until the end of the time horizon or death. Considering the mean baseline age of the patients included and their life expectancy in Spain, a time horizon of 30 years was assumed. A 3% discount rate was applied to health costs and outcomes, in accordance with the Spanish recommendations for economic evaluation and budget impact of drugs [18].

The model estimated the economic and clinical consequences, expressed in quality-adjusted life years (QALY), for each therapeutic alternative during the time horizon. The results of the analysis were evaluated in terms of cost per QALY gained, expressed as the incremental cost-effectiveness ratio (ICER).

### Treatment alternatives evaluated

According to the DECLARE-TIMI 58 trial, the initial cohort included patients with uncontrolled T2DM who had started treatment with dapagliflozin (10 mg/day) or placebo, as add-on therapy to SoC with metformin, sulphonyl urea, insulin, dipeptidyl peptidase-4 (DPP-4) inhibitors, glucagon-like peptide-1 receptor agonists and/or diet [9]. Treatment intensification was not considered, but the model assumed that patients discontinued dapagliflozin at a given annual rate according to the data observed in DECLARE-TIMI 58 trial, and remained on placebo until the end of the time horizon or patient’s death. Additionally, patients discontinued dapagliflozin at an eGFR of 45 ml/min/1.73m^2^.

### Population included and clinical efficacy and safety of treatments

The demographic characteristics and baseline modifiable risk factors of patients with T2DM were obtained from published data from the DECLARE-TIMI 58 trial [9, 17] DAPA-RWE Spain study [19] (Table 1).

**Table 1:** Demographic characteristics and baseline modifiable risk factors of the population included in the model

Variable	Mean (± Standard error)	Distribution	References
**Demographics**			
Age (years)	63.8 (0.052)	Normal	[17]
Female (%)	37 (7.5^a^)	Normal	[9]
Duration of diabetes (years)	10.5 (0.5)	Normal	[9]
Height (m)	1.65 (0.33^a^)	Normal	[19]
**Modifiable risk factors**			
HbA1c (%)	8.3 (0.009)	Normal	[17]
SBP (mmHg)	140.3 (28.1^a^)	Normal	[19]
Weight (kg)	92.0 (18.4^a^)	Normal	[19]
eGFR (ml/min/1.73m^2^)	85.2 (0.15)	Normal	[9]
**CVD History**			
Peripheral artery disease (%)	6.0 (0.1)	Normal	[9]
CHF (%)	5.5 (1.1^a^)	Normal	[20]

^a^Standard error assumed 20% of the mean

*CHF *congestive heart failure, *CVD* cardiovascular disease, *eGFR *estimated glomerular filtration rate, *HbA1c *glycated hemoglobin, *SBP *systolic blood pressure

The efficacy endpoints were the change in glycated hemoglobin (HbA1c), systolic blood pressure (SBP), body weight and eGFR (Table 2). The efficacy of each treatment on modifiable risk factors was applied in the first year, except for the reduction in eGFR that was applied annually. In subsequent years, the model assumed that the progression of HbA1c and SBP was similar to the data observed in the UKPDS study [10], while the progression of weight was considered to be -0.395 kg for dapagliflozin and -0.353 kg for placebo annually according to the DECLARE-TIMI 58 trial [9]. The annual incidence of AEs (diabetic ketoacidosis, urinary and genital tract infection, acute kidney damage and fractures) and severe hypoglycemia, and the discontinuation rate of each treatment from the DECLARE-TIMI 58 trial were included (Table 2) [9, 21].

**Table 2:** Efficacy of treatments

Variable	Dapagliflozin	Placebo	Distribution	Reference
∆ HbA1c (%)	-0.679	-0.151	Normal	[9]
∆ Weight (kg)	-2.415	-0.630	Normal	[9]
∆ SBP (mmHg)	-2.810	-0.409	Normal	[9]
∆ eGFR (ml/min/1.73m^2^)	-1.780	-2.440	Normal	[21]
Adverse events				
Diabetic ketoacidosis	0.0007	0.0003	Normal	[9]
Urinary tract infection	0.0035	0.0037	Normal	[9]
Genital tract infection	0.0021	0.0003	Normal	[9]
Acute kidney failure	0.0035	0.0049	Normal	[9]
Fractures	0.0126	0.0025	Normal	[9]
Severe hypoglycemia	0.0016	0.0023	Normal	[9]
Discontinuation rate	0.049	0.000	Normal	[9]

*eGFR *estimated glomerular filtration rate, *HbA1c *glycated hemoglobin, *SBP *systolic blood pressure

### Costs

According to the perspective used, only direct healthcare costs were included in the analysis. The costs identified were updated to 2021 values based on the healthcare component of the Spanish consumer price index (Table 3).

**Table 3:** Costs related to T2DM (€ 2021)

Parameter	Cost			Distribution	Reference
	First year		Maintenance		
	Fatal event	Non-fatal event			
Micro-and macrovascular complications					
Unstable angina	€ 4557	€ 3090	€ 892	Gamma	[24, 25]
Myocardial infarction	€ 10464	€ 7036	€ 892	Gamma	[24, 25]
Heart failure	€ 4222	€ 3259	€ 3683	Gamma	[24, 25]
Stroke	€ 6 660	€ 4738	€ 3725	Gamma	[24, 25]
End-stage kidney disease	€ 53310	€ 53310	€ 28931	Gamma	[27, 28]
Blindness	-	€ 2275	€ 834	Gamma	[24, 25]
Ulcers	-	€ 5163	€ 402	Gamma	[24, 26]
	**Cost**				**Reference**
Pharmacological treatment					
Dapagliflozin	€ 624.37			Gamma	[22]
Adverse events					
Urinary tract infection	€ 53			Gamma	[22, 29]
Genital tract infection	€ 53			Gamma	[22, 29]
Diabetic ketoacidosis	€ 3942			Gamma	[24]
Acute kidney failure	€ 4151			Gamma	[24]
Fractures	€ 4341			Gamma	[29]
Hypoglycemic events					
Severe hypoglycemia	€ 696			Gamma	[30]
Treatment discontinuation	€ 52			Gamma	[29]
Chronic kidney disease					
Stage 2	€ 1304			Gamma	[31]
Stage 3	€ 4860			Gamma	[32]
Stage 4	€ 8058			Gamma	[33]
Stage 5	€ 13659			Gamma	[33]

#### Pharmacological costs

Dapagliflozin acquisition cost was calculated from the retail price, including value added tax and applying the deduction according to Royal Decree Law 8/2010 (7.5%) [22]. The annual cost of dapagliflozin treatment was estimated at € 624.37 for the recommended dose of 10 mg/day [23].

#### Costs of T2DM complications

The costs of managing micro- and macrovascular complications in T2DM were differentiated in the year of incidence according to whether they were fatal or non-fatal events. In patients who survived, annual maintenance costs were considered for all subsequent years up to the end of the time horizon or patient’s death (Table 3). Costs were obtained from the Spanish Minimum Basic Data Set [24] and from published studies in the Spanish setting [25–28].

#### Costs of AEs, severe hypoglycemic events and treatment discontinuation

The costs of AEs included the costs associated with urinary tract infections, genital tract infections and fractures, and hospitalization costs for diabetic ketoacidosis and acute kidney damage [24]. The cost of managing urinary and genital tract infections included the cost of a primary care physician visit and treatment with amoxicillin [22, 29], while the cost managing fractures was calculated as the mean cost of a fracture of the hip and pelvis, forearm, and humerus [29].

In terms of hypoglycemic events, only the costs of severe hypoglycemic events were considered. These events were calculated from the unit cost of a severe event [30] and the number of events occurred in each cycle.

The cost of treatment discontinuation for dapagliflozin was also considered, assuming the cost of a primary care physician visit [29] (Table 3).

#### CKD-related costs

CKD-related costs were included as the model tracks eGFR progression. The annual cost of each disease stage (2-5) was calculated from Spanish studies [31–33] (Table 3).

### Utilities

The impact of T2DM on health-related quality of life was assessed using utilities and was expressed as QALYs. The baseline utility was 0.800 and was estimated from the European Quality of Life-5 Dimensions questionnaire in an observational study of Spanish T2DM patients [34]. In addition, utility decrements associated with T2DM-related complications, AEs, hypoglycemic events, treatment discontinuation, CKD and body mass index were included (see Additional file 2). Utility values were applied additively and were obtained from published data [35–42] in other settings due to the lack of Spanish data.

### Sensitivity analysis

Univariate sensitivity analyses were performed to evaluate the impact of the parameters on the results of the analysis and to validate their robustness. The parameters modified individually were time horizon (20 years and lifetime), discount rate (0% and 5%), mean baseline age (40 and 70 years), and mean baseline eGFR (70 ml/min/1.73m^2^). These sensitivity analyses were conducted to assess the use of dapagliflozin in different populations (young, older people, with greater renal impairment). When baseline age was varied, time horizon was also modified to simulate the costs and effects proportionally to the base case.

In addition, a probabilistic sensitivity analysis (PSA) was performed, in which the values of all parameters, except patient characteristics, were modified simultaneously in each model run. A cohort of 1,000 patients was simulated over 1,000 runs. A normal distribution was considered for baseline patient characteristics and treatment efficacy, a gamma distribution for costs, and a beta distribution for utilities and probabilities. The analysis assumed a willingness-to-pay (WTP) threshold of € 20,000/QALY gained for Spain [43].

Consolidated Health Economic Evaluation Reporting Standards checklist [44] was applied to ensure a proper reporting of the health economic evaluation (see Additional file 3).

## Results

### Main analysis

Treatment with dapagliflozin was more effective than placebo, resulting in 0.08 more QALYs per patient (10.96 vs. 10.88). Over a 30-year time horizon, dapagliflozin would prevent 17 macrovascular events (495 vs. 512) in a cohort of 1,000 patients, compared with placebo; 17 hospitalizations for heart failure (123 vs. 140), and 7 microvascular events (124 vs. 131).

The prevention of these complications was mainly thanks to a better disease control, with improvements in all modifiable risk factors (HbA1c, body weight, SBP, eGFR) compared with placebo [9] Additionally, dapagliflozin reduced the risk of complications, such as hospitalization for HF and myocardial infarction.

Cost analysis results show that the total cost per patient was € 56,984 with dapagliflozin and € 59,905 with placebo, saving € 2,921 per patient (Table 4). Therefore, the additional acquisition cost of dapagliflozin (€ 4,985) was fully offset by the lower cost of micro- and macrovascular events (€ -7,908) and severe hypoglycemic events (€ -4).

**Table 4:** Base-case and univariate sensitivity analysis results

	Dapagliflozin	Placebo	Difference
**QALYs**	10.96	10.88	0.08
**Total costs (€)**	€ 56,984	€ 59,905	€ -2921
Acquisition of drug	€ 4985	€ 0	€ 4985
T2DM Complications^**a**^	€ 50,916	€ 58,824	€ -7908
Macrovascular	€ 12,959	€ 13,612	€ -653
Microvascular	€ 37,957	€ 45,212	€ -7255
Hypoglycemic events^**b**^	€ 19	€ 23	€ -4
Adverse events^**c**^	€ 1064	€ 1058	€ 6
**ICER (€/QALY)**			Dominant
**Sensitivity analysis**	∆ **Costs**	∆ **QALY**	**ICER (€/QALY)**
Time horizon: lifetime	€ -3590	0.08	Dominant
Time horizon: 20 years	€ -963	0.06	Dominant
Discount rate: 0%	€ -6361	0.12	Dominant
Discount rate: 5%	€ -1553	0.06	Dominant
Baseline age: 40 years	€ -6086	0.10	Dominant
Baseline age: 70 years	€ -992	0.06	Dominant
Baseline eGFR: 70 ml/min/1.73m^**2**^	€ -5120	0.07	Dominant

^a^ Microvascular and macrovascular complications included: unstable angina, myocardial infarction, heart failure, stroke, end-stage kidney disease, blindness, and ulcers

^b^ Only severe hypoglycemic events were included

^c^ The costs of managing urinary and genital tract infections, diabetic acidosis, fractures and acute kidney failure were included

*eGFR *estimated glomerular filtration rate, *ICER *incremental cost-effectiveness ratio, *QALY *Quality-adjusted life year, *T2DM* Type 2 diabetes mellitus

As a result, dapagliflozin was a dominant therapeutic alternative for the treatment of T2DM, resulting in higher effectiveness and lower overall associated costs than placebo.

### Sensitivity analysis

The univariate sensitivity analysis confirmed the robustness of the main analysis. In all scenarios, dapagliflozin was a cost-effective therapeutic option for the treatment of T2DM, considering a WTP threshold of € 20,000/QALY gained (Table 4). A 20-year time horizon and a higher discount rate (5%) reduced slightly the effectiveness of dapagliflozin compared with placebo because long-term benefits of dapagliflozin were not fully captured.

**Fig. 1:**
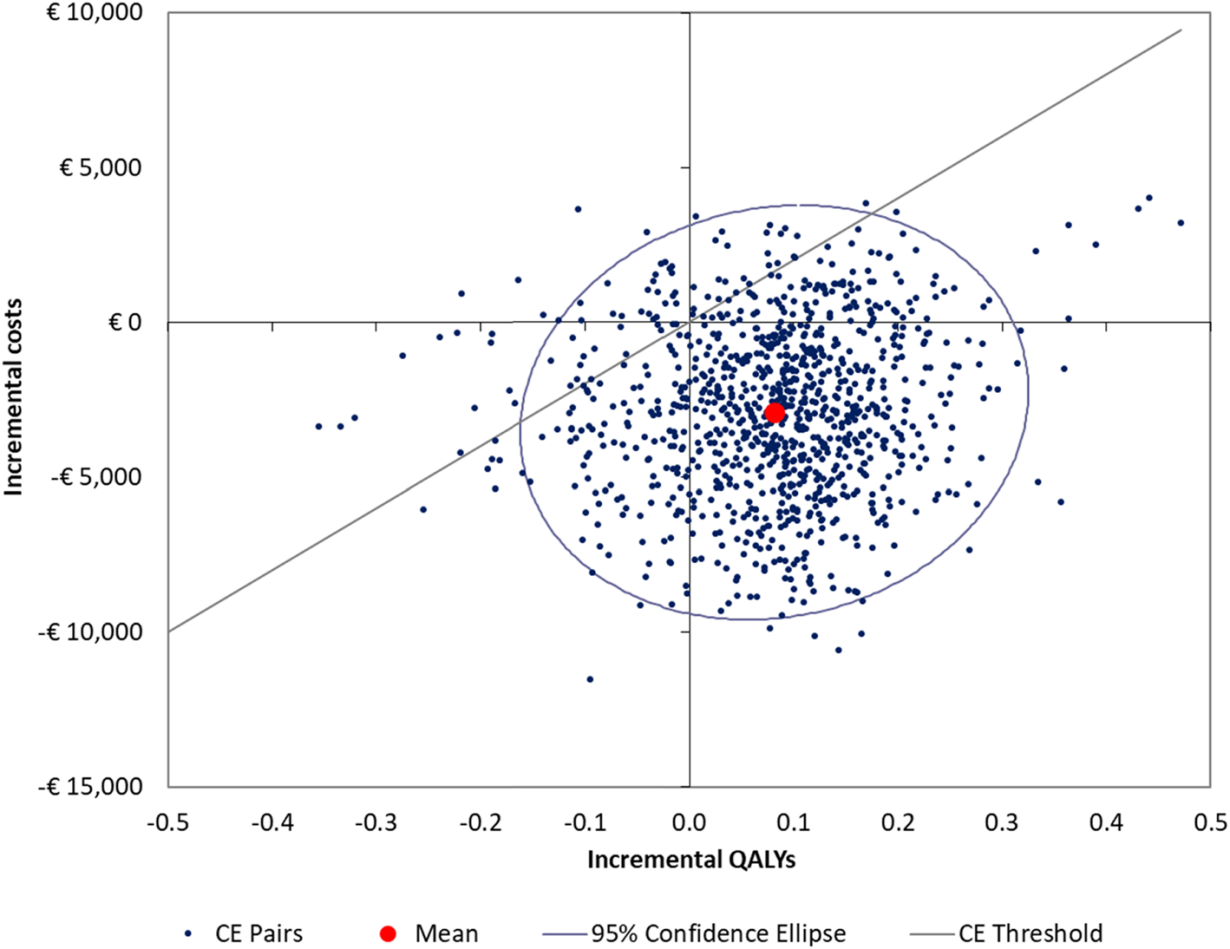
Probabilistic sensitivity analysis. CE: Cost-effectiveness; QALY: Quality-adjusted life year

The PSA showed that dapagliflozin was a dominant option compared with placebo in 84.2% of the simulations and was cost-effective in 92.1% of cases at a WTP threshold of € 20,000/QALY gained (Fig. 1).

## Discussion

The present cost-effectiveness analysis of dapagliflozin based on the data from the DECLARE-TIMI 58 trial show that dapagliflozin is a dominant option compared with placebo in patients with T2DM who had or were at risk of atherosclerotic cardiovascular disease in Spain. Dapagliflozin resulted in increased effectiveness (0.08 QALY) and lower costs (€ -2,921) in the management of T2DM. Dapagliflozin had a beneficial effect on both glycemic control and the reduction of T2DM-related complications, including progression of CKD and hypoglycemic events.

Our findings are similar to those published in an economic evaluation of dapagliflozin performed in the UK, in which the Cardiff T2DM model was also adapted according to the survival results of the DECLARE-TIMI 58 trial [45]. In that study, dapagliflozin was a dominant alternative compared with placebo, resulting in 0.06 more QALYs and cost-savings of £ 2,552. These results were maintained in the subgroup analysis, which evaluated patients with established cardiovascular disease, multiple risk factors, and prior HF, and highlighted the potential of dapagliflozin to reduce the economic burden of T2DM and its associated complications [45].

In a literature review, two economic evaluations of dapagliflozin for the treatment of T2DM were identified in Spain [25, 46]. In one study, dapagliflozin was compared with DPP-4 inhibitors, both in combination with metformin. In line with our study, the results showed that dapagliflozin was a dominant alternative with higher effectiveness (0.019 QALYs) and lower total costs (€ -42) [25]. In the other study, the combination of dapagliflozin and insulin was compared with the combination of DPP-4 inhibitors and insulin, and insulin alone. Dapagliflozin in combination with insulin was a dominant option (0.168 QALYs; € -51) compared with DPP-4 inhibitors and insulin; and it was cost-effective with an ICER of € 2,159/QALY (0.698 QALY; € +1,508) compared with insulin alone [46]. Thus, dapagliflozin was a therapeutic alternative for intensification treatment in T2DM patients with uncontrolled glycemia, which has greater effectiveness than other available options (such as DPP-4 inhibitors and insulin) without a significant economic impact and even making cost savings.

A possible limitation of the present study is long-term extrapolation of data from short-term clinical trials to model disease progression throughout a patient’s lifetime, although this approach is common in most cost-effectiveness models. In addition, the incidence rate of macrovascular events, mortality and ESRD was estimated using the survival equations from the DECLARE-TIMI 58 trial, instead of established risk equations; and, the incidence rate of microvascular events was calculated based on the UKPDS study risk equations, due to the lack of data from the DECLARE-TIMI 58 trial. However, the incidence rate of events was modeled directly using data from the DECLARE-TIMI 58 trial without the need to use surrogate risk markers [9, 45]. Besides, the Cardiff T2DM model used to simulate the progression of T2DM in this analysis has been validated in previous studies as a tool for conducting economic evaluations of new technologies and making health policy decisions [13–16].

A further limitation is related to the patient characteristics used in the model. This analysis assumed that patient profile of the Spanish population with T2DM in clinical practice was similar to that of the patients in the DECLARE-TIMI 58 trial, but published evidence shows that around 38% and 51% of patients treated with dapagliflozin in clinical practice met the inclusion criteria for the DECLARE-TIMI 58 trial [19, 47]. However, univariate sensitivity analyses were carried out, in which baseline characteristics (age and eGFR) were modified, and a PSA were also performed to ensure the representativeness of T2DM patients who may be treated with dapagliflozin in real clinical practice. The results confirmed that dapagliflozin was a cost-effective therapeutic alternative. In addition, real-world evidence with dapagliflozin in other European countries confirmed the improvements in glycemic control and the reduction of cardiovascular and T2DM-related complications observed in the DECLARE-TIMI 58 trial [48–51].

## Conclusions

In conclusion, this analysis suggests that dapagliflozin, as add-on therapy to SoC, is a cost-effective alternative compared with placebo for the treatment of T2DM in patients who had or were at risk of atherosclerotic cardiovascular disease in Spain. Dapagliflozin demonstrated to reduce T2DM-related complications and hypoglycemic events and therefore this study highlights its potential to minimize clinical and economic burden of T2DM.

## Supplementary Information

**Additional file 1:** Validation of the results of the model over a time horizon of 4.2 years with the DECLARE-TIMI 58 trial.

**Additional file 2:** Utilities and utility decrements used in the model.

**Additional file 3:** CHEERS checklist.

## Data Availability

The datasets used and/or analysed during the current study are included in this published article.

## Abbreviations

AEs: Adverse Events
CKD: Chronic Kidney Disease
DM: Diabetes Mellitus
DPP-4: Dipeptidyl Peptidase-4
eGFR: Estimated Glomerular Filtration Rate
ESRD: End-Stage Renal Disease
Hb1Ac: Glycated Hemoglobin
HF: Heart Failure
ICER: Incremental Cost-Effectiveness Ratio
NHS: National Health System
PSA: Probabilistic Sensitivity Analyses
QALY: Quality-Adjusted Life Year
SBP: Systolic Blood Pressure
SoC: Standard of Care
T2DM: Type 2 Diabetes Mellitus
UKPDS: United Kingdom Prospective Diabetes Study
WTP: Willingness-To-Pay
