# Supplementary material for: Cost-effectiveness analysis of dapagliflozin for the treatment of type 2 diabetes mellitus in Spain: results of the DECLARE-TIMI 58 study
Source: BMC Health Serv Res. 2022 Feb 17;22:217. doi: 10.1186/s12913-022-07567-5 (PMC8851809; doi:10.1186/s12913-022-07567-5)
Supplement: Supplementary file 1 — Additional file 1: Validation of the results of the model over a time horizon of 4.2 years with the DECLARE-TIMI 58 trial. [file 12913_2022_7567_MOESM1_ESM.pdf]

Additional file 1. Validation of the results of the model over a time horizon of 4.2 years with the DECLARE-TIMI 58 trial

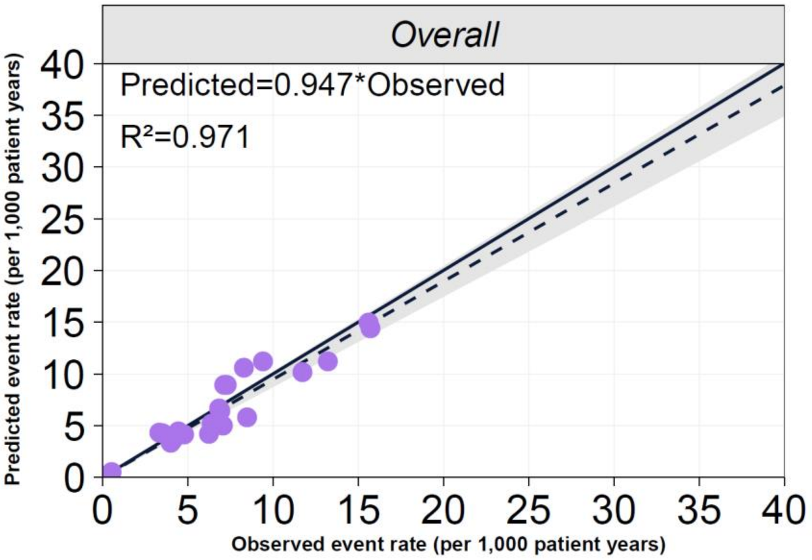

Note: The solid line represents the 45-degree identity line and the dashed line is the fitted linear regression line. The shaded area marks the 95% confidence intervals for the linear regression line.
