# Supplementary material for: Cost-effectiveness analysis of dapagliflozin for the treatment of type 2 diabetes mellitus in Spain: results of the DECLARE-TIMI 58 study
Source: BMC Health Serv Res. 2022 Feb 17;22:217. doi: 10.1186/s12913-022-07567-5 (PMC8851809; doi:10.1186/s12913-022-07567-5)
Supplement: Supplementary file 2 — Additional file 2: Utilities and utility decrements used in the model. [file 12913_2022_7567_MOESM2_ESM.pdf]

Additional file 2. Utilities and utility decrements used in the model

Parameter	Utility	Distribution	Reference
**Baseline utility**	0.800	Beta	[34]
**Micro- and macrovascular complications**			
Unstable angina	-0.042	Beta	[35]
Myocardial infarction	-0.055	Beta	[37]
Heart failure	-0.108	Beta	[37]
Stroke	-0.164	Beta	[37]
End-stage kidney disease	-0.263	Beta	[41]
Blindness	-0.074	Beta	[41]
Ulcers	-0.170	Beta	[42]
**Adverse events**			
Urinary tract infection	-0.003	Beta	[38]
Genital tract infection	-0.003	Beta	Assumption[a]
Diabetic ketoacidosis	-0.047	Beta	Assumption[b]
Acute kidney failure	-0.110	Beta	[35]
Fractures	-0.068	Beta	[35]
**Hypoglycemic events**			
Severe hypoglycemia	-0.047	Beta	[36]
**Treatment discontinuation**	-		Assumption
**CKD**			
Stage 2	0	Beta	[39]
Stage 3	-0.030	Beta	[39]
Stage 4	-0.050	Beta	[39]
Stage 5 (pre-dialysis)	-0.050	Beta	[39]
**BMI**			
Increase of one unit	-0.047	Beta	[40]
Reduction of one unit	+0.017	Beta	[40]

BMI: body mass index; CKD: chronic kidney disease.

[a] Utility decrement was considered to be equivalent to urinary tract infection.

[b] Utility decrement was considered to be equivalent to a severe hypoglycemic event.
