# Supplementary material for: Cost-effectiveness analysis of dapagliflozin for the treatment of type 2 diabetes mellitus in Spain: results of the DECLARE-TIMI 58 study
Source: BMC Health Serv Res. 2022 Feb 17;22:217. doi: 10.1186/s12913-022-07567-5 (PMC8851809; doi:10.1186/s12913-022-07567-5)
Supplement: Supplementary file 3 — Additional file 3: CHEERS checklist. [file 12913_2022_7567_MOESM3_ESM.pdf]

Additional file 3. CHEERS checklist

Section/Item	Item No	Recommendation	Reported on page No/line No
**Title and abstract**			
Title	1	Identify the study as an economic evaluation or use more specific terms such as ‘‘cost-effectiveness analysis’’, and describe the interventions compared.	page 1, lines 1 to 3
Abstract	2	Provide a structured summary of objectives, perspective, setting, methods (including study design and inputs), results (including base case and uncertainty analyses), and conclusions.	pages 2-3, lines 14 to 39
**Introduction**			
Background and objectives	3	Provide an explicit statement of the broader context for the study. Present the study question and its relevance for health policy or practice decisions.	pages 4-5, lines 44 to 79
**Methods**			
Target population and subgroups	4	Describe characteristics of the base case population and subgroups analyzed, including why they were chosen.	page 7, lines 125 to 127; Table 1
Setting and location	5	State relevant aspects of the system(s) in which the decision(s) need(s) to be made.	page 5, lines 73 to 76
Study perspective	6	Describe the perspective of the study and relate this to the costs being evaluated.	page 5, lines 83<br>page 9, lines 147
Comparators	7	Describe the interventions or strategies being compared and state why they were chosen.	page 7, lines 116 to 119
Time horizon	8	State the time horizon(s) over which costs and consequences are being evaluated and say why appropriate.	page 6, lines 107 to 108
Discount rate	9	Report the choice of discount rate(s) used for costs and outcomes and say why appropriate.	page 6, lines 108 to 110
Choice of health outcomes	10	Describe what outcomes were used as the measure(s) of benefit in the evaluation and their relevance for the type of analysis performed.	pages 7, lines 111 to 114
Measurement of effectiveness	11a	Single study-based estimates: Describe fully the design features of the single effectiveness study and why the single study was a sufficient source of clinical effectiveness data.	page 5, lines 67 to 72
	11b	Synthesis-based estimates: Describe fully the methods used for identification of included studies and synthesis of clinical effectiveness data.	not applicable
Measurement and valuation of preference based outcomes	12	If applicable, describe the population and methods used to elicit preferences for outcomes.	not applicable

Estimating resources and costs	13a	Single study-based economic evaluation: Describe approaches used to estimate resource use associated with the alternative interventions. Describe primary or secondary research methods for valuing each resource item in terms of its unit cost. Describe any adjustments made to approximate to opportunity costs.	not applicable
	13b	Model-based economic evaluation: Describe approaches and data sources used to estimate resource use associated with model health states. Describe primary or secondary research methods for valuing each resource item in terms of its unit cost. Describe any adjustments made to approximate to opportunity costs.	pages 10-11, lines 150 to 175
Currency, price date, and conversion	14	Report the dates of the estimated resource quantities and unit costs. Describe methods for adjusting estimated unit costs to the year of reported costs if necessary. Describe methods for converting costs into a common currency base and the exchange rate.	page 9, lines 148 to 149
Choice of model	15	Describe and give reasons for the specific type of decision analytical model used. Providing a figure to show model structure is strongly recommended.	page 5, lines 82 to 87
Assumptions	16	Describe all structural or other assumptions underpinning the decision-analytical model.	page 6, lines 94 to 105<br>page 8, lines 134 to 139
Analytical methods	17	Describe all analytical methods supporting the evaluation. This could include methods for dealing with skewed, missing, or censored data; extrapolation methods; methods for pooling data; approaches to validate or make adjustments (such as half cycle corrections) to a model; and methods for handling population heterogeneity and uncertainty.	page 6, lines 90 to 93<br>page 6, lines 98 to 105
**Results**			
Study parameters	18	Report the values, ranges, references, and, if used, probability distributions for all parameters. Report reasons or sources for distributions used to represent uncertainty where appropriate. Providing a table to show the input values is strongly recommended.	pages 11-12, lines 194 to 196<br>Table 2, Table 3, Additional file 2
Incremental costs and outcomes	19	For each intervention, report mean values for the main categories of estimated costs and outcomes of interest, as well as mean differences between the comparator groups. If applicable, report incremental cost-effectiveness ratios.	Table 4

Characterizing uncertainty	20a	Single study-based economic evaluation: Describe the effects of sampling uncertainty for the estimated incremental cost and incremental effectiveness parameters, together with the impact of methodological assumptions (such as discount rate, study perspective).	not applicable
	20b	Model-based economic evaluation: Describe the effects on the results of uncertainty for all input parameters, and uncertainty related to the structure of the model and assumptions.	page 13, lines 218 to 225<br>Table 4<br>Figure 1
Characteristing heterogeneity	21	If applicable, report differences in costs, outcomes, or cost effectiveness that can be explained by variations between subgroups of patients with different baseline characteristics or other observed variability in effects that are not reducible by more information.	not applicable
**Discussion**			
Study findings, limitations, generalisaiblity, and current knowledge	22	Summarize key study findings and describe how they support the conclusions reached. Discuss limitations and the generalizability of the findings and how the findings fit with current knowledge.	pages 13-14, lines 227 to 236<br>page 14, lines 242 to 254<br>pages 14-15, lines 255 to 278
**Other**			
Source of funding	23	Describe how the study was funded and the role of the funder in the identification, design, conduct, and reporting of the analysis. Describe other non-monetary sources of support	page 17, 309 to 311
Conflicts of interest	24	Describe any potential for conflict of interest of study contributors in accordance with journal policy. In the absence of a journal policy, we recommend authors comply with International Committee of Medical Journal Editors recommendations	page 17, lines 303 to 307
